# Comparative efficacy of gambee and single interrupted suture patterns in reducing complications after canine enterotomy

**DOI:** 10.18849/ve.v10i3.714

**Published:** 2025-07-24

**Authors:** Anthi Anatolitou+, Miltiadis Markou+

**Keywords:** CANINE, COMPLICATED, DOGS, ENTEROTOMY, GAMBEE, SINGLE INTERRUPTED, SUTURE

## Abstract

**PICO question:**

In dogs undergoing enterotomy does using a Gambee suture pattern instead of a single interrupted suture pattern to close the intestinal incision reduce the risk of postoperative complications?

**Clinical bottom line:**

**Category of research:**

Treatment.

**Number and type of study designs reviewed:**

Three papers addressed the PICO question and matched the search terms. All were experimental clinical trials. One study was a controlled but non-randomised trial, another was a non-controlled, non-randomised clinical trial, and the third was a randomised but non-controlled clinical trial.

**Strength of evidence:**

Weak.

**Outcomes reported:**

The first study suggested that the simple interrupted technique was easier, faster, and safer, with a significantly lower stenotic index at the anastomotic site and with relatively lower adhesion formation and rapid gain in tensile strength than the Gambee method. The second study found the risk of postoperative complications after enterotomy in dogs was no different whether Gambee or simple-interrupted sutures were used. The third study showed that the time for closure was significantly less for the simple interrupted suture group compared to the Gambee suture group. Despite this, the mean initial and maximum leak pressure values in the Gambee group were higher.

**Conclusion:**

The study design of those papers is considered poor and the strength of evidence weak in support of the PICO question. For now, the decision between Gambee and single interrupted suture for intestinal closure depends on the vet surgeon’s choice. Future studies should be designed more efficiently before recommending a specific technique in clinical practice.

## How to apply this evidence in practice

The application of evidence into practice should take into account multiple factors, not limited to: individual clinical expertise, patient’s circumstances and owners’ values, country, location or clinic where you work, the individual case in front of you, the availability of therapies and resources.

Knowledge Summaries are a resource to help reinforce or inform decision-making. They do not override the responsibility or judgement of the practitioner to do what is best for the animal in their care.

## Clinical scenario

An 8 months-old female Labrador puppy is referred to your clinic for vomiting and inappetence. The owners believe that their dog probably ingested a missing part of a plastic chewing toy. A radiographic examination of the dog revealed an intestinal foreign body obstruction. An abdominal ultrasound confirmed the intestinal obstruction and you propose to the owners a surgical treatment. The owners are anxious because they previously had another dog that died 5 days after an enterotomy surgery, due to incision dehiscence and peritonitis. They ask you to perform the most effective bowel closure to reduce the risk of postoperative leakage. You consider performing the bowel closure with either a Gambee or a single interrupted suture technique, but are unsure which is more effective at reducing the risk of postoperative leakage and postsurgical complications.

## The evidence

The search criteria identified three studies that answered the PICO question (Athar et al., 1996; Azevedo et al., 2008; Kieves et al., 2018). Two of the papers (Athar et al., 1996; Azevedo et al., 2008) are non-randomised controlled trials while the third is a randomised controlled trial (Kieves et al., 2018). Also, all three papers presented a small sample size, lacked power analysis, and none reported a blinding technique. Only Kieves et al. (2018) referred to randomisation, while Azevedo et al. (2008) did not describe the scoring system they used for macroscopical and morphological evaluation, nor the exact number of samples they registered in each group. Moreover, Kieves et al. (2018) performed only an ex vivo leak pressure comparison in cadaveric tissues without histopathological evaluation. Lastly, Athar et al. (1996) referred to randomisation only in the data analysis, did not mention who performed the sutures, and did not support their results with histopathological examination. In conclusion, all three studies had limitations either in their design or execution that result in weak evidence in support of the PICO question. Consequently, there is weak evidence to recommend a specific suture pattern for canine enterotomy in clinical practice as safer and more efficacious.

## Summary of the evidence

**Athar et al. (1996):** Studies on end-to-end colonic anastomosis in the dog: a comparison of techniques **Aim: **To compare the efficacy of three different end-to-end colonic anastomosis techniques in dogs, focusing on healing quality, lumen patency, and tensile strength.

Population:	Healthy, adult, mongrel dogs of both sexes (Faculty of Veterinary Science, Pakistan).
Sample size:	18 dogs.
Intervention details:	The dogs were categorised into 3 groups: simple interrupted approximating sutures (n = 6); double-layer inverting sutures (n = 6); Gambee sutures (n = 6). All dogs underwent clinical examinations (body temperature, pulses, respiratory rates, blood, urine, and faeces examinations) during the 7-day acclimatisation period. Preoperatively food and water were withheld for 18 and 6 hours, respectively. Premedication with atropine sulphate 0.045 mg/kg of body weight (b.w.) subcutaneously and acepromazine maleate 0.25 mh/kg b.w. intramuscularly, 30 and 15 min respectively before surgery. Induction and maintenance of general anaesthesia with 5% thiopentone sodium intravenously, to effect. All dogs underwent caudal coeliotomy and colonic resection, followed by an end-to-end colonic anastomosis. Postoperatively, water was offered without restriction followed by milk on day 2 and a soft diet on day 3. All dogs were euthanised and had the operated segments of the colon harvested on postoperative day 14.
Study design:	Non-randomised controlled trial.
Outcome Studied:	Observation: Physiological parameters (body temperature, pulse, respiration rates, but not presented extensively) daily up to day 14. Time for return of defaecation (measured in hours). Perianastomotic adhesions, graded as 25%, 50%, 75%, 100% assessed for the extent of circumference involved. Haematological tests: Parameters measured on alternate days up to day 13 (not presented extensively, except of leukocytic response (cells/mm^3^). Stenotic index: Stenotic indices were assessed radiographically, using McAdam’s formula. Gain in tensile strength (TS) of the operated segment of the colon: Tensile strength of the anastomotic site was measured with Schopper’s Tensile Strength Tester No.114-SC type.
Main Findings (relevant to PICO question):	Non-significant difference (P > 0.05) among the three groups for time of normal defaecation (57.33 ± 6.02 to 62.67 ± 6.02 hours). Relatively higher adhesion incidence in Gambee group (91.7 ± 12.9%) compared to the simple interrupted group (87.5 ± 5.6%), postoperatively. However, there was non-significant difference (P ≥0.05) and there was overlap between groups. Significant lesser stenosis at the anastomotic site in the simple interrupted group (26.14 ± 1.87%) compared to theGambee group (30.16 ± 1.20%) at day 14 (P ≤ 0.01). Relatively higher gain in TS in the simple interrupted group (26.55 ± 1.33%) compared to the Gambee group (26.05 ± 0.73%) on day 14. However, there was non-significant difference (P > 0.01) and there was overlap between groups.
Limitations:	Very small sample size. Blinding for time of normal defaecation, haematological, and physiological parameters, gain in tensile strength, adhesion and stenosis incidence were not reported. The p-value was presented as equal to or less than. Suture material and intervals were not mentioned. No information on who performed the surgical procedures was presented. Perianastomotic adhesions grading system was based on rats. Histopathological examinations were not performed, despite it being possible due to the study’s design. Colotomy does not reflect the enterotomy of small intestine. The authors used an experimental model of colonic anastomosis to find the best technique for small intestinal end-to-end anastomosis. Experimental studies do not reflect the clinical cases. Healthy dogs do not reflect the majority of dogs that undergo enterotomy. Short-term study. Time required to complete suturing, or the technical difficulty of each technique were not quantitatively assessed.

**Azevedo et al. (2008):** Comparative study of hand sewn single layer anastomosis of dog’s bowel

Population:	Mongrel dogs (7–12kg). University of Sao Paulo and Graduate Program in Surgery and Experimentation, Brazil.
Sample size:	6 dogs.
Intervention details:	All dogs received two anastomoses: one at 30 cm from de Treitz angle, using a sero-submucosal technique – anteriorly with the anterior sero-submucosal (AS) method and posteriorly with the posterior sero-submucosal (PS) method – and another at 60 cm, using a full-thickness technique – anteriorly with the anterior full-thickness (AT) Gambee suture pattern and posteriorly with the posterior full-thickness (PT) simple interrupted suture pattern. The placement of these techniques was alternated to reduce positional bias.All dogs underwent general anaesthesia with sodium pentobarbital 30 mg/kg. All dogs underwent medial laparotomy and each dog received two anastomoses. All anastomoses were performed with single stitches at 3 mm intervals using blue monofilament polypropylene 4-0, 2 cm needle. No antibiotics were given to the dogs postoperatively and food was offered without restriction. All dogs were euthanised on day 7 postoperatively and the anastomotic sites were harvested.
Study design:	Non-randomised clinical trial.
Outcome Studied:	Macroscopic evaluation: Peritoneal adhesions over the suture line of anastomosis. Microscopic evaluation: Proliferation and wider inflammation assessed using morphometry.
Main Findings (relevant to PICO question):	No significant difference between the peritoneal adhesion scores over the suture line of anastomosis of AT and PT groups (P < 0.01). No statistical difference between morphometry of inflammation (macrophages, neutrophils, fibroblasts and collagen fibers) in the AT and PT groups (P < 0.01).
Limitations:	Very small sample size. The number of samples assigned to each technique group was not specified. Age and sex of the dogs were not presented. Blinding for macroscopic and microscopic analysis was not reported. The trial was non-randomised. No information on who performed the surgical procedures was presented. Enterotomy of small intestine does not reflect the enterotomy of large intestine. Experimental studies do not reflect the clinical cases. Healthy dogs do not reflect the majority of dogs that undergo enterotomy. Suture material was nonabsorbent. Research took place over a short timeframe.

**Kieves et al. (2018):** A comparison of ex vivo leak pressures for four enterotomy closures in a canine model **Aim: **To compare the leak resistance and closure time of four enterotomy closure techniques in a canine ex vivo model to assess their effectiveness and clinical applicability.

Population:	Healthy, adult (26.5 ± 9.3 months old), intact female, (13.6 ± 3.5 kg) beagle dogs (College of Veterinary Medicine, Iowa).
Sample size:	12 dogs.
Intervention details:	Forty-eight 3 cm enterotomy constructs from jejunal segments which were first harvested and divided into four 14 cm segments; these constructs were randomly assigned to the four closure pattern groups; modified Gambee (n = 12), simple interrupted (n = 12), simple continuous (n = 12), and skin staple (n = 12). All dogs were euthanised with pentobarbiral-phenytoin sodium intravenously and the entire jejunum was harvested. A 3 cm enterotomy was performed along the antimesenteric border. All enterotomies were sutured with 3-0 polydioxanone small half-circle (SH) 26 mm tapered needle, placed at 2–3 mm intervals and 2–3 mm apart from the incision edge. A single person performed all closures.
Study design:	Randomised clinical trial.
Outcome Studied:	Time: Time in seconds to perform the stitches was recorded. Leak pressure: Initial and maximum leak pressure was measured.
Main Findings (relevant to PICO question):	There was significant difference (P < 0.05) between the mean (± standard deviation) closure time in seconds for the simple interrupted pattern (262.2 ± 46.5 seconds) and the modified Gambee pattern (356.1 ± 60 seconds). No significant difference (P > 0.01) between the initial leak pressure of the modified Gambee (50.7 ± 15.4 mm Hg) and the simple interrupted pattern (38.6 ± 15.9 mm Hg) No significant difference (P > 0.01) between the maximum leak pressure of the modified Gambee (123.7 ± 28.8 mm Hg) and the simple interrupted group (81.4 ± 40.4 mm Hg). However, there was an overlap of the mean initial leak pressures between the two groups. In the modified Gambee group most of the failures (83%) occurred only at the suture hole, while for the simple interrupted group at the suture hole and the incision place.
Limitations:	Small sample size. Blinding for time and leak pressure analysis were not reported. Histopathological examinations were not performed. Experimental studies do not reflect the clinical cases. Insufficient data for the failures in the simple interrupted group. Ex vivo study does not reflect an in vivo study. Enterotomy of small intestine does not reflect the enterotomy of large intestine Handling of the tissue was not evaluated, due to the study’s design.

## Appraisal, application and reflection

Enterotomy is the most commonly performed surgery on the small intestine, due to intestinal obstruction, trauma, malpositioning, and diagnostic procedures (Brown, 2012). Intestinal healing depends on blood supply, tissue apposition, and minimal surgical trauma (Hedlund & Fossum, 2007). Among other postoperative complications, dehiscence is the most devastating. In one retrospective study of 121 dogs, Allen et al. (1992) found that the rate of dehiscence after enterotomy or enteroanastomosis was 19/121 (15.7%), which resulted in a mortality rate of 14/19 (73.7%). Obstruction by foreign bodies augmented these rates at 10/38 (26%) and 11/13 (85%), respectively (Mullen et al., 2020). As a result, ongoing research in this field focuses on exploring various suture techniques and materials to identify an approach that maximises advantages while minimising disadvantages. A comparative cohort study of 214 dogs revealed no difference between hand suture and stapled enteroanastomosis techniques, regarding the frequency of dehiscence (Duell et al., 2016). On the contrary, DePompeo et al. (2018) presented that end-to-end stapled anastomosis was less likely to result in dehiscence compared to sutured closure. There is still a lack of evidence for the most suitable suture pattern for intestinal closure in dogs.

All the studies appraised in this Knowledge Summary were experimental case studies (Athar et al., 1996; Azevedo et al., 2008; Kieves et al., 2018), which compared the safety and efficacy of different suture patterns for enteroanastomosis in dogs. Two of the papers were conducted in vivo (Athar et al. 1996; Azevedo et al. 2008) and examined anastomotic techniques for the large and small intestines, respectively. The study by Athar et al. (1996) analysed many variables in dogs that underwent three different end-to-end colonic anastomoses. This study revealed no statistical difference between the Gambee and the simple interrupted technique group on day 14, except for the mean stenotic index. Also, relatively higher adhesion formation scores (91.7 ± 12.9%) and lower gain in the tensile strength (26.05 ± 0.73%) were found in the Gambee group compared to the single interrupted group (87.5 ± 5.6%, 26.55 ± 1.33% respectively). However, there was a significant amount of overlap between groups. Specifically, the authors suggested that a simple interrupted suture is more suitable for colonic anastomosis. Moreover, they found that simple interrupted sutures induced relatively lesser perianastomotic adhesions and gained relatively higher tensile strength in comparison to Gambee sutures. However, relative comparisons are unlikely to be reliable when seeking strong outcomes, and despite the design of the study, no histological tests were performed. On the contrary, Azevedo et al. (2008) performed two intestinal anastomoses in each dog, one sero-submucosal at 30 cm from de Treitz angle and one total technique at 60 cm. They aimed to compare four different single-layer patterns using polypropylene sutures. On the seventh postoperative day, the dogs were euthanised and the anastomotic sites were examined both microscopically and macroscopically. The authors concluded that the adhesion scores and the morphological features were similar among simple interrupted and Gambee groups. Lastly, the final experimental case study, examined the time of closure and the ex vivo leak pressures for jejunum anastomosis (Kieves et al. 2018). Although the outcomes demonstrated the efficacy of the modified Gambee suture compared to skin staples – showing significantly higher maximum leak pressures – the study found no significant difference between the Gambee and simple interrupted groups in terms of initial and maximum leak pressures. Despite the higher p-value (P ≤ 0.5) the article’s authors concluded that Gambee closure was the slowest to perform. Additionally, most leakages were noticed at the suture holes in the Gambee pattern group (83%), while in the simple interrupted technique group, leakages were mentioned both at the suture holes and the incision line. Note that the ex vivo design of the study did not allow histopathological evaluation and further examinations relevant to the PICO question.

All three studies had several limitations that weakened their evidence. All studies lacked power analysis for sample estimation, had a very small sample size and no control groups. All studies reported blinding during the examinations. Also, randomisation was only noted by Kieves et al. (2018), while Athar et al. (1996) reported randomisation only for data analysis. Furthermore, Azevedo et al. (2018) did not describe the number of animals in each group nor the scoring system they used to evaluate macroscopic and microscopic parameters, such as adhesion formation or tissue healing. Additionally, the age of the animals was not recorded in all studies, and this could interfere with the total results. Only Kieves et al. (2018) referred to adult, intact female Beagle dogs, but the exact age was not presented. Older dogs may be more likely to have concurrent diseases (like cancer, endocrinological diseases, etc.) that might decrease intestinal restoration. Similarly, histopathological analysis was only reported by Azevedo et al. (2018). Generally, enterotomies may be more likely to be performed into abnormal tissue so histopathological analysis could reveal much more information. However, the ex vivo study of Kieves et al. (2018) did not allow microscopical analysis.

Moreover, Athar et al. (1996) and Azevedo et al. (2008) did not mention who did the statistical analysis or the surgical procedures, and Kieves et al. (2018) did not report who recorded the time needed for closures. Accordingly, if the authors conducted them, a systematic error could be introduced in their study. Also, if the surgeries were not performed by the same person, this may affect the results, as an experienced vet surgeon might need less time and cause less tissue handling compared to a newly qualified one. An experienced veterinary surgeon might complete the procedure more quickly and handle tissues more gently than a newly qualified one, potentially resulting in reduced inflammation and adhesion formation. Another source of bias in the study by Athar et al. (1996) was the reliance on relative comparisons between groups, with many outcomes based on non-significant differences. The authors did not note that those results were subjective and interpretive. As a result, the overall conclusions appeared subjective, particularly since aspects such as suture simplicity and time required for completion were not statistically examined. Relative comparisons without statistical significance be misleading and should not be used as the basis for definitive conclusions. reported as evidence. Lastly, only Kieves et al. (2018) used a monofilament, absorbable suture material. On the contrary, Azevedo et al. (2008) used a nonabsorbable suture, while Athar et al. (1996) did not report either the suture material or the intervals. Usually, nonabsorbable sutures are not used for enteroanastomosis or intestinal closure, because if they extrude into the intestinal lumen they could act as possible sites for attachment of newly ingested foreign bodies (Milovancev et al., 2004). Nevertheless an absorbable suture may be more likely to initiate an inflammatory response compared to a nonabsorbable suture, while larger intervals between stitches might more easily permit postoperative leakage.

Furthermore, all three studies were experimental studies that do not reflect the clinical scenario of the PICO. Specifically, healthy dogs do not reflect the majority of dogs that undergo enterotomy. The majority of dogs that need enterotomy are more likely to have unbalanced biochemical values (like hypoalbuminemia, hypokalemia, etc.), higher stress and concurrent illness that may affect negatively the healing process. Both Azevedo et al. (2008) and Kieves et al. (2018) studied anastomosis of the small intestine, while Athar et al. (1996) investigated the healing of the large intestine. Enterotomy of the small intestine does not reflect the enterotomy of the large intestine. The small intestine has a small bacterial population and higher healing rates, while the colon has a decreased capacity to lay down collagen which make more likely postoperative leakage and sepsis Recognising these differences, Athar et al. (1996) conducted a controlled experimental study, which offers numerous benefits, such as the ability to establish causal relationships by isolating specific variables and minimising confounding factors. Controlled studies provide high internal validity, ensuring that outcomes are directly linked to the intervention. Positioned near the top of the evidence pyramid, just below systematic reviews and meta-analyses, they play a critical role in supporting reliable clinical guidelines and decision-making.

An equally important limitation was was the short follow-up period in both the Athar et al. (1996) and Azevedo et al. (2008) studies, as animals were euthanised shortly after surgery. Usually, dogs that undergo enterotomies may be more likely to have long-term postoperative complications, like intestinal obstruction due to adhesions, strictures, and other causes. Finally, Kieves et al. (2018) used cadaveric tissue instead of live tissue which may interfere with the outcomes. The normal peristaltic movements and the passage of food in living dogs, add more pressure on the intestinal lumen.

In the surgical field, the decision between Gambee and suture interrupted suture for intestinal closure depends on the vet surgeon’s choice. Until stronger evidence emerges, the vet surgeon must decide which suture pattern works better for them according to their experience. For instance, the use of a Gambee suture may require more time and experience. Also, it will likely necessitate more tissue handling and be difficult to perform especially in small dogs. According to Athar et al. (1996), simple interrupted suture resulted in lesser stenosis of the bowel lumen, lesser adhesion formation and rapid gain in tensile strength compared to Gambee. On the contrary, Kieves et al. (2018) found that leak failures occurred for Gambee sutures at the suture hole, while for simple interrupted sutures leakage occurred both at the suture hole and the incision line. This is indicative that Gambee suture might be safer and more resistant, but more research is needed. Finally, Azevedo et al. (2008) claimed that sero-submucosal sutures, which extend to the submucosa and are tied over the serosa were more appropriate for enterotomy closure compared to total sutures regarding inflammation, adhesion and regeneration of the tissue.

In conclusion, directly comparing the available studies is challenging due to their distinct characteristics, including differences in study models (in vivo versus ex vivo), suture patterns, materials, anastomosis sites, and perioperative protocols. The heterogeneity among the studies – particularly between the two in vivo models – limits the ability to draw unified conclusions. While the Gambee suture has shown some promising results, the current body of evidence remains limited and insufficient to confidently recommend any specific suture pattern as the safest choice for intestinal closure in clinical practice. To advance clinical decision-making, future research should focus on well-designed randomised controlled trials with standardised methodologies, larger sample sizes based on power analysis, and robust statistical analyses. Such studies should compare commonly used suture patterns in terms of complication rates, healing outcomes, and long-term efficacy, ultimately supporting more reliable, evidence-based recommendations.

## Methodology

**Search Strategy:** 

Databases searched and dates covered:	CAB Abstracts on CABI: 1973 to November 2024 PubMed via NCBI: 1920 to November 2024
Search strategy:	CAB Abstracts: (dog or dogs or canine* or canis).mp. or exp dogs/ (intestin* or enterotom* or surg* or operat* or incision*).mp. ((Gambee or 'single interrupted' or 'simple interrupted') and (suture* or swaged or pattern or patterns or stitch* or clos* or technique or techniques or ligature or ligatures or method or methods)).mp. (postoperative* or post-operative* or postsurgical* or post-surgical* or postincisional* or post-incisional*).mp. and ((complic* or problem* or disorder or disorders or dehiscence or paralytic ileus or stricture* or avulsion or incarceration).mp. or exp complications/) 1 and 2 and 3 and 4 PubMed: dog OR dogs OR canine OR canines OR canis intestine OR enterotomy OR surgery OR operation OR incision (Gambee OR 'single interrupted' OR 'simple interrupted') AND (suture OR swaged OR pattern OR patterns OR stitch OR closure OR technique OR techniques OR ligature OR ligatures OR method OR methods) (postoperative OR post-operative OR postsurgical* or post-surgical* OR postincisional* OR post-incisional*) AND (complication OR problem OR disorder OR dehiscence OR paralytic ileus OR stricture OR avulsion OR incarceration) 1 and 2 and 3 and 4
Dates searches performed:	29 Nov 2024

**Exclusion / Inclusion Criteria:** 

Exclusion:	Duplicates. Does not answer the PICO question.
Inclusion:	Study design relevant to primary research (experimental studies, case series, clinical trials).

**Search Outcome:** 

Database	Number of results	Excluded – duplicates	Excluded – did not answer the PICO question	Excluded – Study design not relevant to primary research	Total relevant papers
CAB Abstracts	41	0	39	0	2
PubMed	54	15	38	0	1
Total relevant papers when duplicates removed					3

## ORCiD

Anthi Anatolitou: https://orcid.org/0000-0002-8705-3249

Miltiadis Markou: https://orcid.org/0000-0001-9754-2057

## Conflict of Interest

The author declares no conflicts of interest.
